# *Prevotella* abundance and salivary amylase gene copy number predict fat loss in response to wholegrain diets

**DOI:** 10.3389/fnut.2022.947349

**Published:** 2022-08-22

**Authors:** Lars Christensen, Mads F. Hjorth, Lukasz Krych, Tine Rask Licht, Lotte Lauritzen, Faidon Magkos, Henrik M. Roager

**Affiliations:** ^1^Department of Nutrition, Exercise and Sports, University of Copenhagen, Copenhagen, Denmark; ^2^Novo Nordisk Foundation, Copenhagen, Denmark; ^3^Department of Food Science, University of Copenhagen, Copenhagen, Denmark; ^4^National Food Institute, Technical University of Denmark, Kongens Lyngby, Denmark

**Keywords:** weight loss, obesity, enterotypes, microbiota, *Prevotella*, AMY1, wholegrains, dietary fiber

## Abstract

**Background:**

Salivary amylase (AMY1) gene copy number (CN) and *Prevotella* abundance in the gut are involved in carbohydrate digestion in the upper and lower gastrointestinal tract, respectively; and have been suggested as prognostic biomarkers for weight loss among overweight individuals consuming diets rich in fiber and wholegrains.

**Objective:**

We hypothesized that *Prevotella* abundance would be linked to greater loss of body fat after wholegrain consumption among individuals with low AMY1 CN, but not in those with high AMY1 CN.

**Methods:**

We reanalyzed data from two independent randomized *ad libitum* wholegrain interventions (fiber intake ∼33 g/d for 6–8 weeks), to investigate the relationship between baseline *Prevotella* abundance and body fat loss among healthy, overweight participants stratified into two groups by median AMY1 CN. Individuals with no detected *Prevotella* spp. were excluded from the main analysis.

**Results:**

In both studies, individuals with low AMY1 CN exhibited a positive correlation between baseline *Prevotella* abundance and fat loss after consuming the wholegrain diet (*r* > 0.5, *P* < 0.05), but no correlation among participants with high AMY1 CN (*P* ≥ 0.6). Following consumption of the refined wheat control diets, there were no associations between baseline *Prevotella* abundance and changes in body fat in any of the AMY1 groups.

**Conclusion:**

These results suggest that *Prevotella* abundance together with AMY1 CN can help predict fat loss in response to *ad libitum* wholegrain diets, highlighting the potential of these biomarkers in personalized obesity management.

## Introduction

*Ad libitum* diets rich in wholegrain (WG) have been found to reduce body weight compared with diets rich in refined grains (1–3). However, there is a large inter-individual variation in the weight loss success upon WG consumption. Given the role of the gut microbiota in digesting dietary polysaccharides (4), it is likely that individual differences in gut microbiota explain some of this variation in weight loss (5). Accordingly, we demonstrated that participants with high abundance of *Prevotella* lost more weight than participants with low abundance of *Prevotella* after 6 weeks of WG consumption (6), and that this loss could be associated with change in appetite regulation (7). Besides dietary fiber, WG is rich in starch, which is degraded enzymatically in humans by salivary amylase; the enzymatic ability to degrade starch differs considerably from individual to individual, as the copy number (CN) of the salivary amylase gene (AMY1) has been shown to vary from 1 to 27 (8). Consequently, AMY1 CN influences the amount of starch that remains undigested and made available to gut microbes (9), and can therefore influence the composition and function of gut microbial populations. For instance, participants with low AMY1 copy numbers have enhanced gut microbiome capacity to break down polysaccharides (10) and produce more methane (11). AMY1 CN has also been associated - both positively and negatively – with adiposity (12, 13), and we recently found that weight loss after consumption of a plant and fiber-rich diet for 26 weeks was positively associated with baseline *Prevotella*-to*-Bacteroides* ratio in overweight participants with low AMY CN, but not in participants with high AMY CN (14).

Here, we investigated the interaction between baseline *Prevotella* abundance and AMY1 CN in predicting weight loss and body fat loss in two independent but similar WG intervention trials. We hypothesized that *Prevotella* abundance would be linked to greater loss of body weight and fat after *ad libitum* intake of WG for 6–8 weeks in healthy, overweight participants with low AMY1 CN, but not in those with high AMY1 CN.

## Materials and methods

Both WG intervention studies, study 1 [Vuholm et al. (15)] and study 2 [Roager et al. (2)], were conducted in accordance with the 1975 Declaration of Helsinki guidelines at the Department of Nutrition, Exercise, and Sports, University of Copenhagen, Denmark, in 2013 and 2014 respectively.

### Study designs

Study 1: As described in Vuholm et al. (15), 75 healthy overweight adults were enrolled in a randomized, controlled, researcher-blinded, parallel 6-week trial. Subjects were assigned to either WG (rye and wheat) or refined wheat (RW) diets. At weeks 0 and 6, subjects collected a spot fecal sample, which was analyzed to determine gut microbiota; body composition measurements were performed by Dual-energy X-ray absorptiometry (DEXA). Study 2: As described in Roager et al. (2), 60 healthy overweight adults were enrolled in a randomized, controlled cross-over trial with two 8-week periods (WG diet or RW diet in random order, separated by a 6-week washout). Examinations were conducted at the beginning and the end of each period and included collection of a spot fecal sample, and body composition measurements performed by bioelectrical impedance analysis (QuadScan 4000, Bodystat Inc., Isle of Man, British Isles, United Kingdom).

### Intervention diets

In both studies, diets were consumed *ad libitum* with no caloric restriction, and participants were instructed to replace all cereal products in their habitual diet with the provided WG products (while the control group received refined grain products). At baseline and during the last week of the interventions, participants completed a 4-day food record with a registration of the type and amount of all foods and beverages that they had consumed. Dietary compliance was evaluated by measuring plasma alkylresorcinols, as previously described (2, 15).

### Fecal microbiota composition

Study 1: The microbiota composition of pretreatment fecal samples was analyzed by 16S rRNA gene (V3–V4 region) MiSeq-based (Illumina Inc.) sequencing. The Greengenes (version 13.8, Quantitative Insight Into Microbial Ecology) 16S rRNA gene collection was used as a reference database, and QIIME (versions 1.7.0 and 1.8.0) was used for analyzing the sequenced data with subsampling at 9,500 reads/sample, as described previously (15). Study 2: The pretreatment microbiota composition was analyzed by shotgun metagenomic sequencing with an average of seven gigabases readout per sample (Illumina 100 bp pair end), and the microbial sequences obtained from shotgun metagenomics were mapped to the integrated catalog of reference genes of the human gut microbiome (i.e., integrated gene catalog), as previously described (2). In both studies, genus-level baseline log-transformed *Prevotella* abundance was used as a surrogate of microbial enterotype (16). Subjects with no detected *Prevotella* spp. were excluded from the main analyses (but included in a secondary analysis), as previous studies indicate that these subjects respond differently than subjects with high and low *Prevotella* abundance, respectively, when consuming fiber-rich diets (6, 7).

### Salivary amylase gene copy number

In both studies, the copy number variation of the AMY1 locus was analyzed from human buffy coat samples using droplet digital polymerase chain reaction (ddPCR), as previously described (14). Copy numbers of AMY1 were analyzed using QuantaSoft software version 1.7.4 (Bio-Rad Laboratories), assuming a diploid nature for the EIF2C1 reference gene. The median values of AMY1 CN was used as the cutoff to define low and high AMY1 CN groups in study 1 (median = 6.8) and study 2 (median = 6.4).

### Statistics

Statistical analyses were conducted in R [R Core Team, (17)], version 3.4.2 (R Foundation). For each study, differences in baseline characteristics between the AMY1 groups were evaluated using an unpaired two sample *t*-test (normally distributed data), Wilcoxon rank-sum test (non-normally distributed data), and Pearson’s chi-square test (categorical data). Normality was assessed by visual inspection of residual plots, histograms and normal probability plots. Data not normally distributed including fecal microbiome data were log transformed before analysis. Correlations between baseline log-transformed *Prevotella* abundance and changes in body weight and fat (difference between endpoint and baseline) were analyzed by means of Pearson’s correlation coefficients in R and using the *ppcor* package when controlling for baseline body weight or baseline body fat, respectively. After combining the two studies, a linear multiple regression model was applied to assess the relationship between baseline log-transformed *Prevotella* abundance and change in body fat percentage while adjusting for baseline body fat percentage and study. Plots were created in R, version 3.4.2, and GraphPad Prism, version 8.3.1. The level of significance was set at *P* < 0.05.

## Results

**FIGURE 1:**
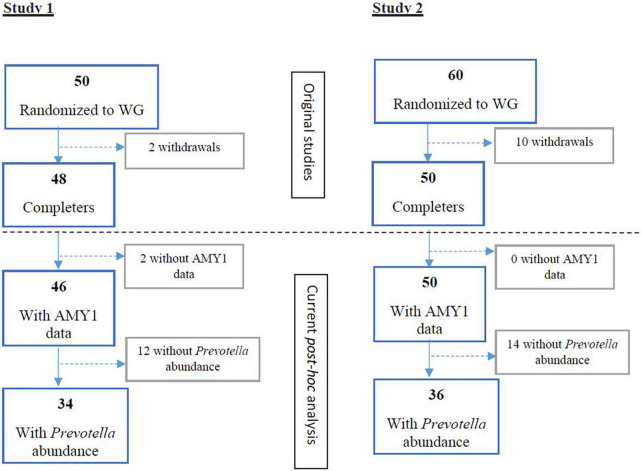
Flow of participants randomized to wholegrain (WG) diets and with baseline *Prevotella* abundance and AMY1 gene copy number data for study 1 (15) and study 2 (2). WG, wholegrain.

In study 1 [Vuholm et al. (15)] and 2 [Roager et al. (2)], 34 and 36 participants with detectable *Prevotella* abundance at baseline completed the WG interventions, respectively (Figure 1). Among these participants, AMY1 CN ranged from 2 to 12 (Supplementary Figures 1, 2). After stratifying participants by median AMY1 CN for each study, no differences in baseline characteristics, including body weight, body fat percentage, and *Prevotella* abundance were found between high and low AMY1 CN groups (Supplementary Tables 1, 2). During the *ad libitum* WG interventions, participants consumed on average 33 g (±11.4 g) of dietary fiber for 6 weeks (study 1) and 33 g (±10.0 g) of dietary fiber for 8 weeks (study 2).

### Body fat loss is influenced by salivary amylase gene copy number and *Prevotella* abundance

**FIGURE 2:**
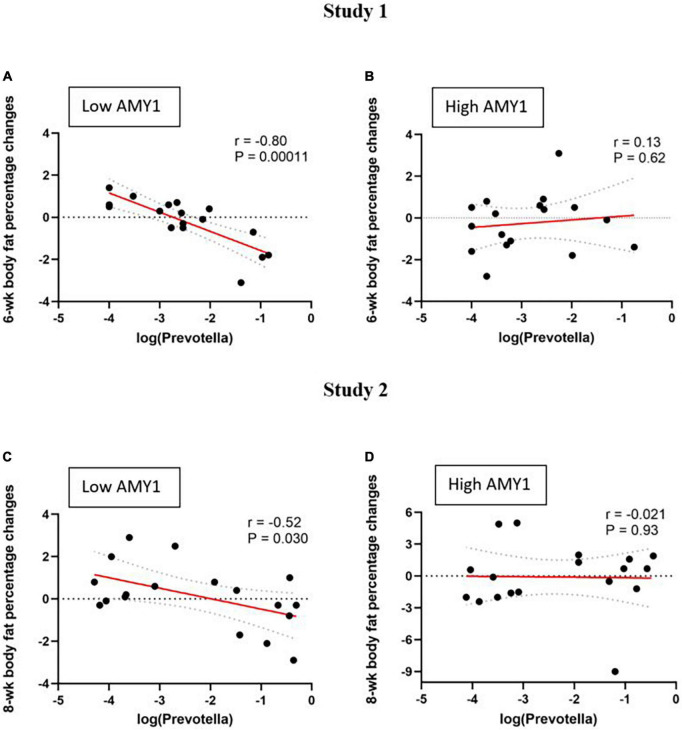
Correlations between baseline *Prevotella* abundance and change in body fat percentage (difference between endpoint and baseline) during two different wholegrain interventions in people with low and high AMY1 gene copy number. Study 1 (*n* = 34): **(A)** low AMY1 (*n* = 17) and **(B)** high AMY1 (*n* = 17) groups; and Study 2 (*n* = 36): **(C)** low AMY1 (*n* = 18) and **(D)** high AMY1 (*n* = 18) groups. Pearson’s correlation coefficients (r) and corresponding *P*-values are shown. Linear regression are depicted in solid red lines and respective 95% confidence intervals are drawn in dashed lines.

Among participants with low AMY1 CN in study 1 (*n* = 17), baseline *Prevotella* abundance correlated with 6-wk changes in body weight (*r* = −0.59, *P* = 0.014), body fat mass (*r* = −0.72, *P* = 0.0012), and body fat percentage (*r* = −0.80, *P* = 0.0001) (Figure 2A). The association between *Prevotella* abundance and body fat percentage was also evident after controlling for baseline body fat percentage (*P* = 0.0002). On the contrary, no such relationship was observed among those with high AMY1 CN (*n* = 17, *P* ≥ 0.6) (Figure 2B).

Similarly, among low AMY1 CN participants in study 2, we found a relationship between *Prevotella* abundance and 8-wk changes in body fat percentage (*n* = 18, *r* = −0.52, *P* = 0.030) (Figure 2C). This association was also evident after controlling for baseline body fat percentage (*P* < 0.04). Again, this association was not observed among the high AMY1 participants (*n* = 18, *P* ≥ 0.9) (Figure 2D). Also, in study 2, fat mass loss following WG consumption tended to correlate with baseline *Prevotella* abundance among the low AMY1 participants (*r* = 0.42, *P* = 0.08); however, weight loss did not correlate with *Prevotella* abundance in either AMY1 groups (*P* > 0.05).

When the studies were combined, we confirmed that baseline *Prevotella* abundance predicted change in body fat percentage (β = −0.59, *P* = 0.0005) among the participants with low AMY1 CN (*n* = 35) by multiple linear regression adjusted for baseline body fat percentage and study, while there was no relationship among the ones with high AMY1 CN (*n* = 35; β = −0.07, *P* = 0.84).

Notably, in agreement with our previous results (6), no correlations were observed between baseline *Prevotella* abundance and body weight and fat change in any of the studies when including the participants with no detectable *Prevotella* at baseline (Supplementary Figures 3, 4). Furthermore, none of the studies showed any correlations between baseline *Prevotella* abundance and changes in body weight and fat mass measures following consumption of the RW diets in any of the AMY1 groups (data not shown).

## Discussion

Salivary amylase gene gene copy number and *Prevotella* abundance in the gut are both implicated in plant polysaccharide digestion in the upper and lower gastrointestinal tract, respectively (9, 18). We previously observed in study 1 that participants with greater *Prevotella* abundance lose more body weight in response to an *ad libitum* WG dietary intervention (6). Despite relatively small groups, we here found that combining *Prevotella* abundance with AMY1 CN leads to improved prediction of weight loss responses following WG consumption. More specifically, we found that the association between *Prevotella* abundance and fat loss was observed only among participants with low AMY1 CN. To validate this finding, we analyzed data from another independent WG intervention trial (2). Again, we showed that *Prevotella* abundance was positively correlated to fat loss among participants with low AMY1 CN. In both intervention studies, we found no associations between *Prevotella* and fat loss in the high AMY1 groups, or in either AMY1 group in response to the control RW-rich diets. Collectively, three independent studies in healthy, overweight Danish adults now link baseline *Prevotella* abundance to weight or fat loss success in individuals with low AMY1 participants consuming a fiber-rich diet (≥33 g/d) (14). However, it should be noted that Hjorth et al. (14) investigated a varied plant-based (New Nordic) diet with an even greater fiber intake (∼42 g/d), while the two present studies supplied WG rye and wheat products with 33 g/d of fibers and high abundance of arabinoxylan (AX) fibers. Previous studies suggest that *P. copri*, the main species of *Prevotella* (14), but also distinct *Bacteroides* species, are specialized in degrading AX fibers (19, 20). Yet, the functional capacity to degrade AX by *P. copri* depends on the abundance of different *P. copri* “clades” (21), which is a limitation in studies on the potential link between the abundance of the genus *Prevotella* and weight loss. Accordingly, this underlines the need to resolve microbial species and clades in future studies.

When considering that roughly 25–50% of caloric intake of typical diets comes from starch (22), and that the ability of the human host to degrade starch varies tremendously (8), it is likely that interactions between starch and amylase in the upper gastrointestinal tract have a substantial impact on glucose and body weight regulation, but also gut microbiota composition (8, 10–12, 23). With low amylase secretion due to low AMY1 CN, more starch from WG may escape digestion and propagate to the colon and thereby increase total availability of polysaccharide (9). This could potentially result in increased colonic fermentation depending on the individual’s gut microbiota composition and its capacity to metabolize starch. Besides dietary fiber, *Prevotella* has been linked to fermentation of starch in the gut (18), which lead to production of short-chain fatty acids (SCFAs) (24). SCFAs have been shown to promote production of appetite-regulating hormones in the gut, and may also enter the systemic circulation and affect adipose tissue and the brain (7).

In agreement with previous analyses (6, 25), we found individuals with no detectable *Prevotella* abundance at baseline independent of whether 16S rRNA sequencing (study 1) or shotgun metagenomics sequencing (study 2) were applied. Future studies will need to clarify whether these subjects do in fact have a distinct microbiota composition from low and high *Prevotella* subjects, or whether *Prevotella* in those subjects is simply below the detection limit of the sequencing methodologies (7). Other limitations of the current *post-hoc* analyses include the small sizes of the groups after stratification for AMY1 CN, and the relatively short duration of the studies, which is not optimal when investigating changes in body weight and body composition. Nonetheless, we analyzed changes in body fat, rather than only changes in body weight as done in previous similar studies (6, 20) in order to get insight into the tissue composition of the lost weight. In fact, we observed that body fat loss exhibited a stronger correlation with *Prevotella* abundance than body weight loss.

In conclusion, baseline *Prevotella* abundance was associated with greater fat loss in response to *ad libitum* wholegrain consumption among participants with low AMY1 CN, but not among participants with high AMY1 CN. This suggests that both baseline abundance of *Prevotella* and AMY1 CN may be used as biomarkers for predicting weight and fat loss responses to diets rich in fiber and wholegrains.

## Data availability statement

The original contributions presented in this study are included in the article/Supplementary material, further inquiries can be directed to the corresponding author.

## Ethics statement

The studies involving human participants were reviewed and approved by the Ethical Committee of the Capital Region of Denmark. The patients/participants provided their written informed consent to participate in this study.

## Author contributions

LC, MFH, and HR conceived the idea and designed the *post-hoc* analysis. LC performed lab work, analyzed data, and drafted the manuscript and had primary responsibility for the final content. All authors revised the manuscript critically for important intellectual content, and approved the final version.

## Abbreviations

AMY1: salivary amylase gene
CN: copy number
SCFAs: short-chain fatty acids
RW: refined wheat
WG: wholegrain.
