# Supplementary material for: Prevotella abundance and salivary amylase gene copy number predict fat loss in response to wholegrain diets
Source: Front Nutr. 2022 Aug 22;9:947349. doi: 10.3389/fnut.2022.947349 (PMC9441811; doi:10.3389/fnut.2022.947349)
Supplement: Supplementary file 1 [file Data_Sheet_1.PDF]

**Title:** *Prevotella* abundance and salivary amylase gene copy number predict fat loss in response to wholegrain diets. *Christensen et al.*

## Online Supplementary Material

**Supplemental Table 1:** Baseline characteristics of healthy, overweight completers with *Prevotella* abundance stratified by median AMY1 CN (n = 34) from study 1 (1)

| **Study 1** | Low AMY1 CN (1.9 – 6.7) (n = 17) | High AMY1 CN (6.9 – 12.1) (n = 17) | P value |
|---|---|---|---|
| Age, y | 52.4 ± 8.9 | 49.6 ± 10.5 | 0.41 |
| Female sex, n (%) | 11 (64.7 %) | 9 (52.9 %) | 0.49 |
| Body weight, kg | 84.0 ± 9.6 | 84.5 ± 7.0 | 0.87 |
| Body fat, kg | 30.6 ± 3.5 | 29.3 ± 6.3 | 0.47 |
| Body fat, % | 36.5 ± 4.8 | 34.5 ± 7.3 | 0.35 |
| *Prevotella*, RA | 0.0027 (0.0049, 0.085) | 0.0006 (0.0002, 0.0055) | 0.40 |
| *Bacteroides*, RA | 0.032 (0.013, 0.068) | 0.019 (0.0049, 0.085) | 0.55 |

Data are presented as means ± SDs, median (interquartile range), or proportions. *n* = 34. Differences between the AMY1 CN groups were tested using an unpaired 2-sample t-test, Wilcoxon rank-sum test [when data reported as median (IQR)], or Pearson chi-squared test. AMY1, salivary amylase gene; CN, copy number. RA, relative abundance.

**Supplemental Table 2:** Baseline characteristics of healthy, overweight completers with *Prevotella* abundance stratified by median AMY1 CN (n = 36) from study 2 (2)

| **Study 2** | Low AMY1 CN (3.8 – 6.3) (n = 18) | High AMY1 CN (6.5 – 11.4) (n = 18) | P value |
|---|---|---|---|
| Age, y | 45.8 ± 12.2 | 52.1 ± 10.3 | 0.10 |
| Female sex, n (%) | 9 (50 %) | 13 (72.2 %) | 0.17 |
| Body weight, kg | 90.4 ± 13.2 | 83.2 ± 14.0 | 0.12 |
| Body fat, kg | 29.1 ± 9.4 | 27.9 ± 9.8 | 0.71 |
| Body fat, % | 32.1 ± 9.3 | 33.3 ± 8.8 | 0.70 |
| *Prevotella*, RA | 0.0071 (0.00021, 0.19) | 0.0065 (0.00032, 0.087) | 0.99 |
| *Bacteroides*, RA | 0.055 (0.023, 0.11) | 0.057 (0.038, 0.067) | 0.96 |

Data are presented as means ± SDs, median (interquartile range), or proportions. *n* = 36. Differences between the AMY1 CN groups were tested using an unpaired 2-sample t-test, Wilcoxon rank-sum test [when data reported as median (IQR)], or Pearson chi-squared test. AMY1, salivary amylase gene; CN, copy number; RA, relative abundance.

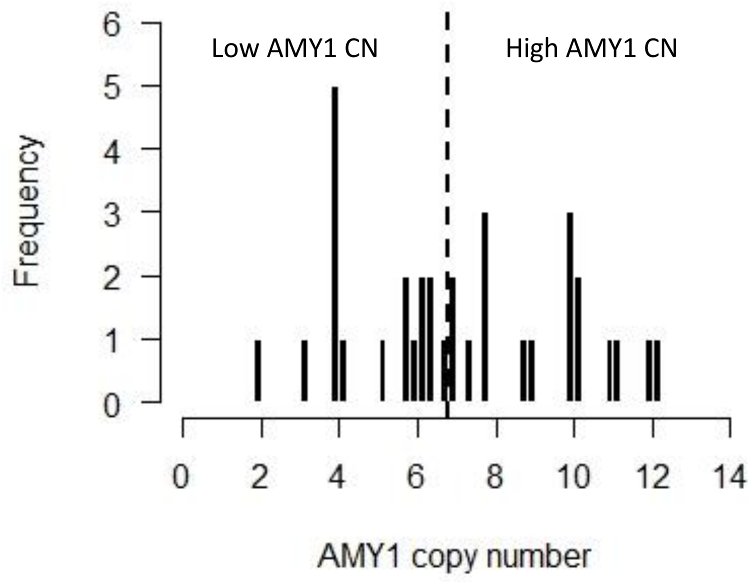


**Supplemental Figure 1:** Histogram showing the distribution of AMY1 copy numbers for healthy, overweight completers with *Prevotella* abundance ($n$ = 34) in study 1 (1). The vertical, dotted line marks the median AMY1 CN (6.8).

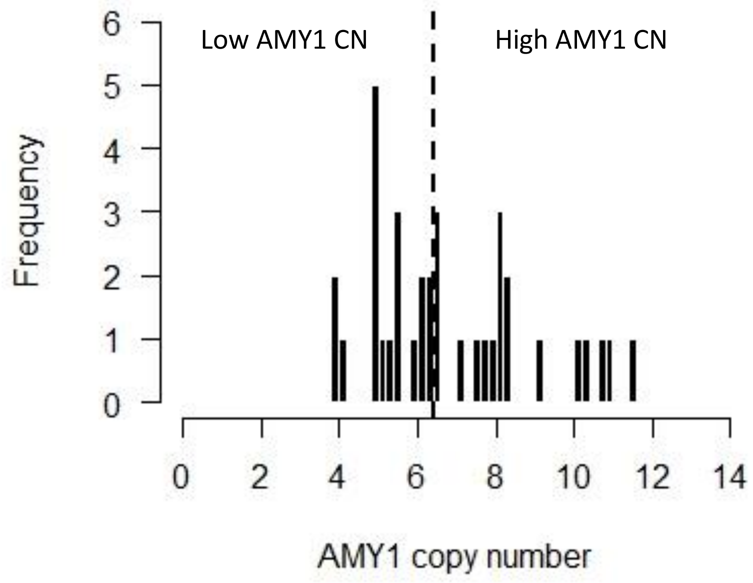


**Supplemental Figure 2:** Histogram showing the distribution of AMY1 copy numbers for healthy, overweight completers with *Prevotella* abundance ($n$ = 36) in study 2 (2). The vertical, dotted line marks the median AMY1 CN (6.4).

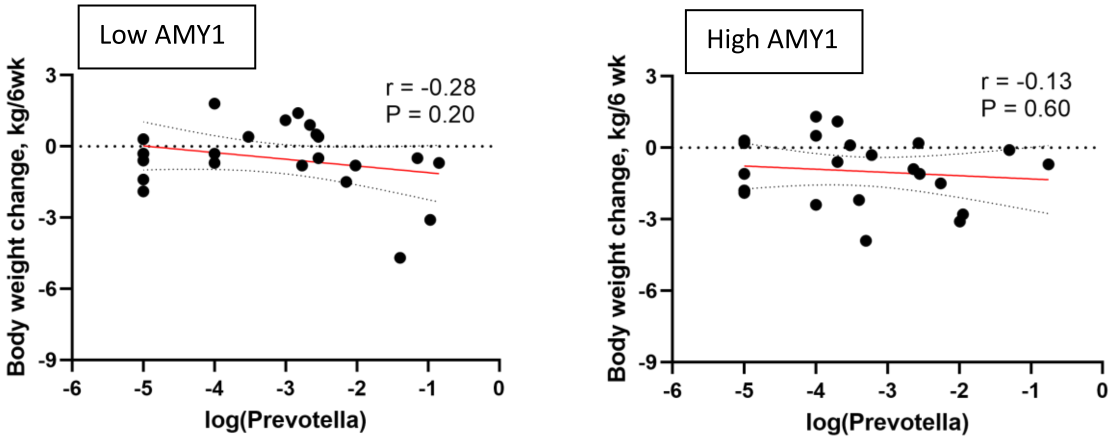


**Supplemental Figure 3:** Relationship between *Prevotella* abundance and body weight change among completers from **study 1,** including the subjects where *Prevotella* was not detected at baseline (n = 12, assigned x value = -5), stratified by median AMY1 gene CN (6.9) into high (n = 23) and low AMY1 (n = 23) groups, total n = 46. Pearson's correlation r and p values are shown.

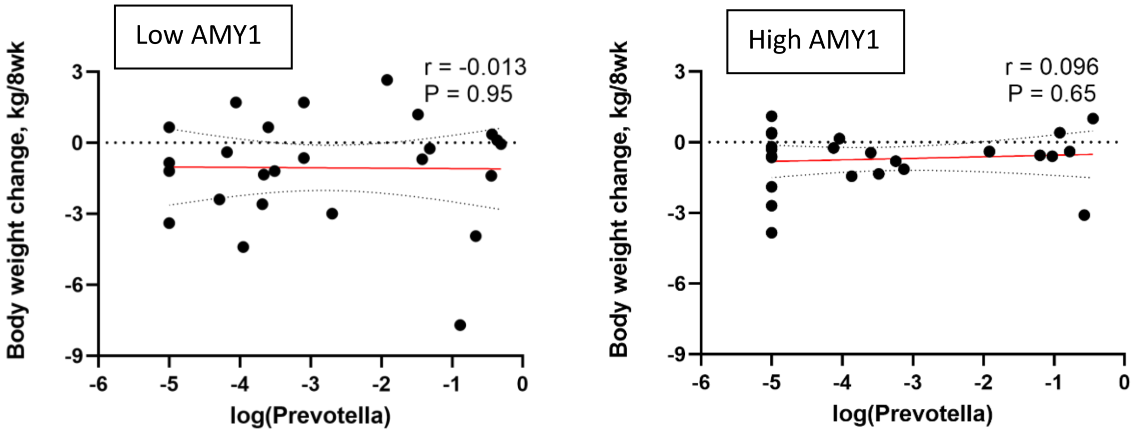


**Supplemental Figure 4:** Relationship between *Prevotella* abundance and body weight change among completers from **study 2,** including the ones where *Prevotella* was not detected at baseline (n = 14, assigned x-value = -5), stratified by median AMY1 gene CN (6.7) into high and low AMY1 groups, total n = 50; (**A**) low AMY1 (n = 25) and (**B**) high AMY1 (n = 25) groups. Pearson's correlation r and p values are shown. Linear regression are depicted in solid red lines and respective 95% confidence intervals are drawn in dashed lines.

## References:

1) Vuholm S, Nielsen DS, Iversen KN, Suhr J, Westermann P, Krych L, Andersen JR, Kristensen M. Whole-Grain Rye and Wheat Affect Some Markers of Gut Health without Altering the Fecal Microbiota in Healthy Overweight Adults: A 6-Week Randomized Trial. J Nutr 2017;147:2067–75.
2) Roager H, Vogt JK, Kristensen M, Hansen LBS, Ibrügger S, Maerkedahl RB, Bahl MI, Lind MV, Nielsen RL, Frøkiaer H, et al. Whole grain-rich diet reduces body weight and systemic low-grade inflammation without inducing major changes of the gut microbiome: A randomised cross-over trial. Gut 2019; 68:83–93.
